# Lexical data for the historical comparison of Rgyalrongic languages

**DOI:** 10.12688/openreseurope.16017.1

**Published:** 2023-06-20

**Authors:** Yunfan Lai, Johann-Mattis List

**Affiliations:** 1 Department of Linguistic and Cultural Evolution, Max Planck Institute for Evolutionary Anthropology, Leipzig, 04103, Germany; 2 Trinity Centre for Asian Studies, The University of Dublin Trinity College, Dublin, Leinster, D02 PN40, Ireland; 3 Multilingual Computational Linguistics, Universitat Passau, Passau, Bavaria, 94032, Germany

**Keywords:** lexical data; historical linguistics; language phylogeny; Rgyalrongic; Sino-Tibetan; language subgrouping; partial cognate annotation; endangered languages

## Abstract

As one of the most morphologically conservative branches of the Sino-Tibetan language family, most of the Rgyalrongic languages are still understudied and poorly understood, not to mention their vulnerable or endangered status. It is therefore important for available data of these languages to be made accessible. The present lexical data sets provide comparative word lists of 20 modern and medieval Rgyalrongic languages, consisting of word lists from fieldwork carried out by the first author and other colleagues as well as published word lists by other authors. In particular, data of the two Khroskyabs varieties are collected by the first author from 2011 to 2016. Cognate identification is based on the authors' expertise in Rgyalrong historical linguistics through the neogrammarian comparative method. We curated the data by conducting phonemic segmantation and partial cognate annotation. The data sets can be used by historical linguists interested in the etymology and the phylogeny of the languages in question, and they can use them to answer questions regarding individual word histories or the subgrouping of languages in this important branch of Sino-Tibetan.

## Plain language summary

Rgyalrongic languages are mainly spoken in Western Sichuan, China, albeit including a now extinct, medieval language, namely Tangut, attested in today’s Ningxia Province. For most non-native speakers, they are the most difficult branch of languages to learn in the Sino-Tibetan family, as they exhibit a large array of word formation strategies such as inflection and derivation. Their word complexity points to their old age and their value in finding out the origin of Sino-Tibetan. This database aims at gathering lexical information of Rgyalrongic languages, in preparation for future work on historical and evolutionary linguistics.

## Introduction

Rgyalrongic languages are a Sino-Tibetan branch, mainly spoken in Rngaba Tibetan and Qiang Autonomous Prefecture in Sichuan, China ^1, 2^. They are closely related to Lolo-Burmese languages ^3^. Apart from their modern varieties, which are mostly endangered or vulnerable, a medieval language, Tangut, is recently recognised to belong to said branch ^4^. Rgyalrongic languages are traditionally divided in two sub-branches, the east sub-branch and the west sub-branch. East Rgyalrongic is comprised of four main languages, Situ, Zbu, Japhug and Tshobdun, and West Rgyalrongic of three further sub-branches, Khroskyabs, Stau (aka. Daofu) and Tangut. Recent phylogenetic studies, however, show that Zhaba is also clustered in Rgyalrongic ^3^. Thus, it is very likely that language varieties closely linked to Zhaba, such as Queyu and Minyag (aka. Muya, Menya) and Zlarong spoken in the Tibetan Autonmous Region, are also Rgyalrongic languages. These “newcomers” are provisionally termed “Peripheral Rgyalrongic” in the following.

**Figure 1.**
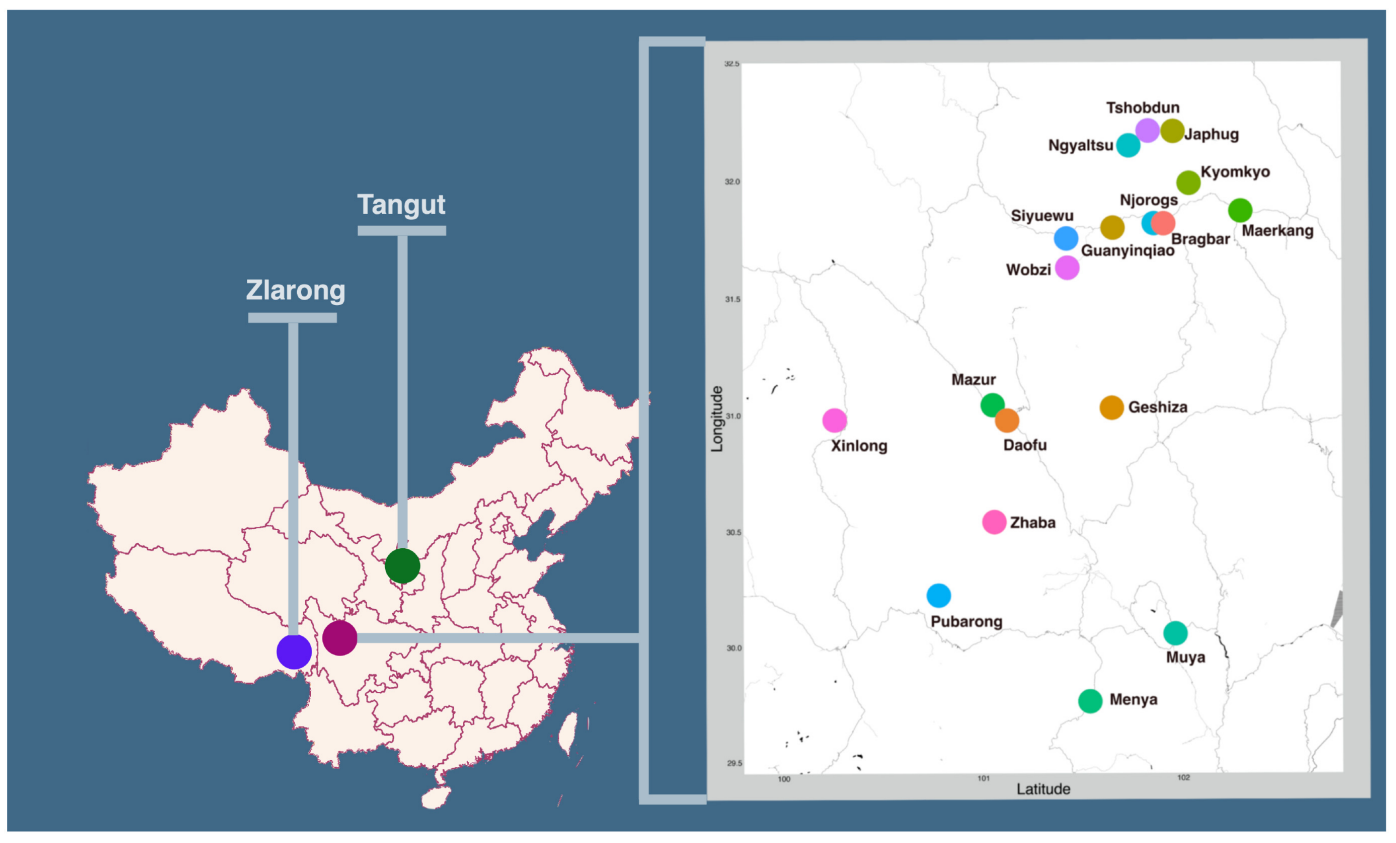
Geographical distribution of the languages in the database.

Rgyalrongic is one of the most morphologically conservative branches in the Sino-Tibetan family, and certainly the closest to Proto-Sino-Tibetan compared to all other varieties in China. Therefore, understanding the history of Rgyalrongic languages is vital for the study on the evolution of Sino-Tibetan. The phylogeny of Rgyalrongic with dating information is thus an essential step towards this goal. Lexical data is the most accessible means to approach language phylogeny and has been proven to show accurate results ^3, 5^. In order to infer the phylogenetic subgrouping of Rgyalrongic, a lexical dataset with high quality curation is inevitable. This database provides the first annotated resource for the phylogenetic analyses of Rgyalrongic languages. It includes twenty varieties in East, West and Peripheral Rgyalrongic, as shown in the map in Figure 1 and Table 1.

**Table 1.** Languages selected for the database.

Languages ID	Language name	Glottolog	Longitude	Latitude	Source
Bantawa	Kiranti Bantawa	bant1281	87.05	27.12	Doornebal (2009) ^6^
Daofu	rGyalrong Daofu	horp1240	101.12	30.98	Huang (1992) ^7^
Japhug	rGyalrong Japhug	japh1234	101.96	32.21	Jacques (2015) ^8^
Tangut	Tangut	tan1334	106.29	38.48	Li (1997) ^9^
WobziKhroskyabs	Khroskyabs Wobzi	eree1240	101.43	31.63	Lai (2017) ^10^
Zhaba	Qiangic Zhaba	zhab1238	101.06	30.54	Huang (1992) ^7^
MaerkangrGyalrong	rGyalrong Maerkang	situ1238	102.30	31.87	Huang (1992) ^7^
MuyaKangding	Muya-kangding	west2417	101.96	30.06	Huang (1992) ^7^
MenyaGao	Menya-Gao	west2417	101.53	29.80	Gao (2015) ^11^
OldBurmese	OldBurmese	oldb1235	96.60	21.46	Dictionary
QueyuXinlong	Queyu Xinlong	quey1238	100.26	30.98	Huang (1992) ^7^
QueyuPubarong	Queyu Pubarong	quey1238	100.78	30.22	Guan Xuan’s fieldwork
SiyuewuKhroskyabs	Siyuewu Khroskyabs	siya1242	101.42	31.75	Author’s fieldwork
BragbarSitu	Bragbar Situ	situ1238	101.91	31.82	Zhang (2020) ^12^
Geshiza	Geshiza	horp1240	101.65	31.03	Honkasalo (2019) ^13^
GuanyinqiaoKhroskyabs	Guanyinqiao Khroskyabs	guan1252	101.66	31.78	Huang (2007) ^14^
NjorogsKhroskyabs	Njorogs Khroskyabs	yelo1242	101.86	31.82	Yin (2007) ^15^
Tshobdun	Tshobdun	tsho1240	101.83	32.21	Sun (2019) ^16^
NgyaltsuZbu	Ngyaltsu Zbu	zbua1234	101.74	32.15	Gong (2018) ^17^
Zlarong	Zlarong	zlar1234	98.09	29.93	Zhao (2019) ^18^
KyomkyoSitu	Kymokyo Situ	situ1238	102.04	31.99	Prins (2017) ^19^
MazurStau	MazurStau	daof1238	101.05	31.04	Gates (2021) ^20^

## Methods

**Figure 2.**
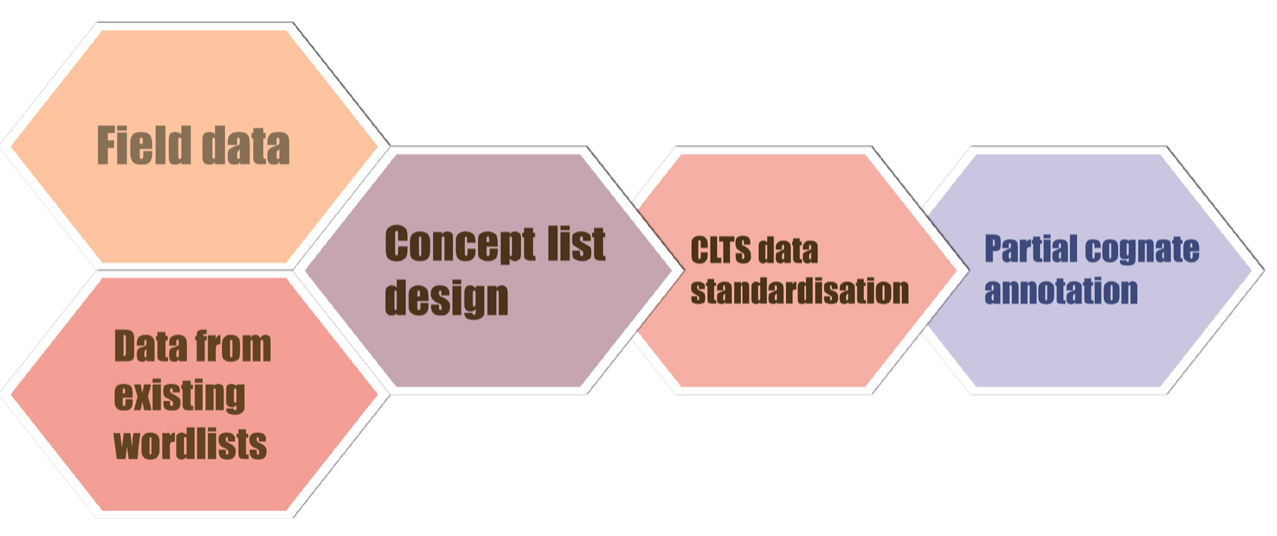
Workflow of building up the Rgyalrongic lexical database.

The entire workflow to build our database is illustrated in Figure 2. We started with the collection of raw data, collected from original fieldwork and from existing word lists. We then organised our raw data into a specifically designed and curated word list. In the third step, we conducted data standardisation conforming to standards outlined by the Cross-Linguistic Data Formats Initiative. Finally, we identified and annotated cognate sets for individual morphemes, also known as *partial cognates* ^21^.

### Major sources of the dataset

The major sources of the dataset include original fieldwork from one of the authors of this study (YFL) and various colleagues who generously shared their lexical data, ie. word lists. The author’s original field data involves two varieties of Khroskyabs, Siyuewu and Wobzi. All the vocabulary needed for this dataset has already been collected before 2017, prior to any of the research projects acknowledged in this paper. Fieldwork involved verbal exchanges with native speakers, requesting pronunciations of words and expressions, without physical contact. An additional source of data was published dictionaries and word lists which were judged as reliable by the authors (see Table 1). Dictionaries and word lists typically contain word forms and translations in Chinese, French or English. Some word lists also provide morphological information and example sentences. Reliability is assessed through two aspects: i) internal phonological consistency of the source data. The authors check if phonemes are correctly identified and that allophones conditioned by phonological environments and morphological alternations are adequately represented in the original sources. ii) external regularity of sound correspondences with the comparative method. The authors checked if cognate forms in the sources exhibit regular correspondences or correspondences that can potentially be explained through alternations or analogy. Languages in our dataset are listed with their sources, their Glottocodes ^22^ and approximate coordinates in Table 1. There are several cases where two or three languages share the same Glottocode (for the general idea behind Glottocodes, see Forkel and Hammarström ^23^). Maerkang (MaerkangrGyalrong), Bragbar Situ (BragbarSitu) and Kyomkyo Situ (Kyomkyositu) share the glottocode “situ1238”, however, they are distinct varieties of Situ with limited intelligibility. The two dialects of Minyag sharing the glottocode “west2417”, labelled MuyaKangding and MenyaGao, are closely related dialects with minor differences. Queyuxinlong and QueyuPubarong (quey1238) are closely related dialects with significant differences in phonology and vocabulary.

Apart from Rgyalrongic languages, we have included two outgroup Sino-Tibetan languages for the accuracy of phylogenetic inference: Bantawa and Old Burmese. The outgroup is used as a reference point to locate and root the ingroup (Rgyalrongic languages). Bantawa belongs to the Kiranti branch mainly spoken in Nepal. Old Burmese was an ancient Lolo-Burmese language attested between 12th and 16th century in present day Myanmar. These two languages are remotely related to Rgyalrongic. According to Sagart *et al.* ^3^, Kiranti languages branched off from other Sino-Tibetan subgroups approximately 5500 years from present, and Lolo-Burmese separated from Rgyalrongic some 4300 years from present. These two languages are suitable for outgroups in the present study, as Bantawa is sufficiently remote to Rgyalrongic, and Old Burmese has a clear date of attestation and can be used for the calibration of dating.

### Data presentation

An extended concept list based on the one used in Sagart *et al.* ^3^ is employed as a guideline of our word selection in each language, including 313 concepts linked to Concepticon ^24^ which provides a unique identifier to all concepts and thus largely facilitates language documentation and historical comparison of lexicon. The concept list used is specially designed for Sino-Tibetan languages, therefore it is most suitable as the starting point of the present dataset. According to our data quality and coverage, we made minor modifications to that concept list by adding and deleting some of the concepts. In particular, we added concepts having a wide coverage in Rgyalrongic languages which are however not widely distributed from the perspectives of the entire Sino-Tibetan family. For instance, we use the general concept for ‘person, human’ instead of ‘the man (male human)’ used in Sagart *et al.* ^3^, which has been shown to be indicative of language subgrouping by Lai ^25^; we also included ‘girl’, as a significant innovation in West Gyalrongic with an *s-*prefix (compare Stau (West) *s-mi* and Japhug *me* (East)), discussed in Lai *et al.* [ 4, 177]. In addition, concepts such as ‘knife’, ‘work’ and ‘sit’ and so on also exhibit similar types of innovations across Rgyalrongic languages, we therefore consider them as worthwhile to be included in the dataset.

### Data standardisation

After collecting the raw word list of each language, we conducted a standardisation process of the data, because the original phonetic transcriptions may differ from each other, some may not adhere strictly to the rules of the International Phonetic Alphabet. The revised transcriptions are based on the transcription conventions in Cross-Linguistic Data Formats reference catalog (CLTS, https://clts.clld.org, 21, 26– 28) and set up an orthography profile ^29^ that helped us automatically convert all transcriptions according to our standard. The standardised data aims specifically at the computation of language phylogeny.

### Partial cognate annotation

Cognates are words or part of words in different languages that share the same origin, such as English *foot* and German *fuß*, both originating from Proto-Germanic *fōts. Cognate forms in daughter languages can be deducted through regular sound rules from the proto-form. In Sino-Tibetan languages, more often than not, we find cognates in word parts rather than in entire words. For instance, Mandarin Chinese *yuè-liang* ‘moon’ and Taiwanese Southern Min *guēh-niû ^n^* ‘moon’ only share the first part *yuè* and *guēh*, respectively, as cognates. This type of cognacy is termed “partial cognacy”. Partial cognates enable us to segment word forms into morphemes, which improves the accuracy of the computation of language subgrouping. It is thus essential to annotate partial cognates, rather than full cognates, in our Rgyalrongic database. Partial cognate identification is conducted manually with the expert knowledge of the authors, using the web-based EDICTOR tool ( https://digling.org/edictor, 30, 31).

### Statistics

The current dataset contains a total of 6335 word forms for 22 distinct language varieties. Word forms correspond to 305 different concepts, and use a total of 413 distinct sounds, with an average inventory size of 72 different sounds per language variety. The word forms have been morphologically segmented, comprising a total of 9116 morphemes. These morphemes have been assigned to 3109 cognate sets. Of these cognate sets, 1665 are unique, which means that we could not identify related words in other languages.

### Quality control

We carefully verified the data to ensure the accuracy and correctness. We use our expert knowledge to review every lexical entry in the database. Whenever in doubt, we would contact the authors of the original sources for confirmation and correction.

**Figure 3.**
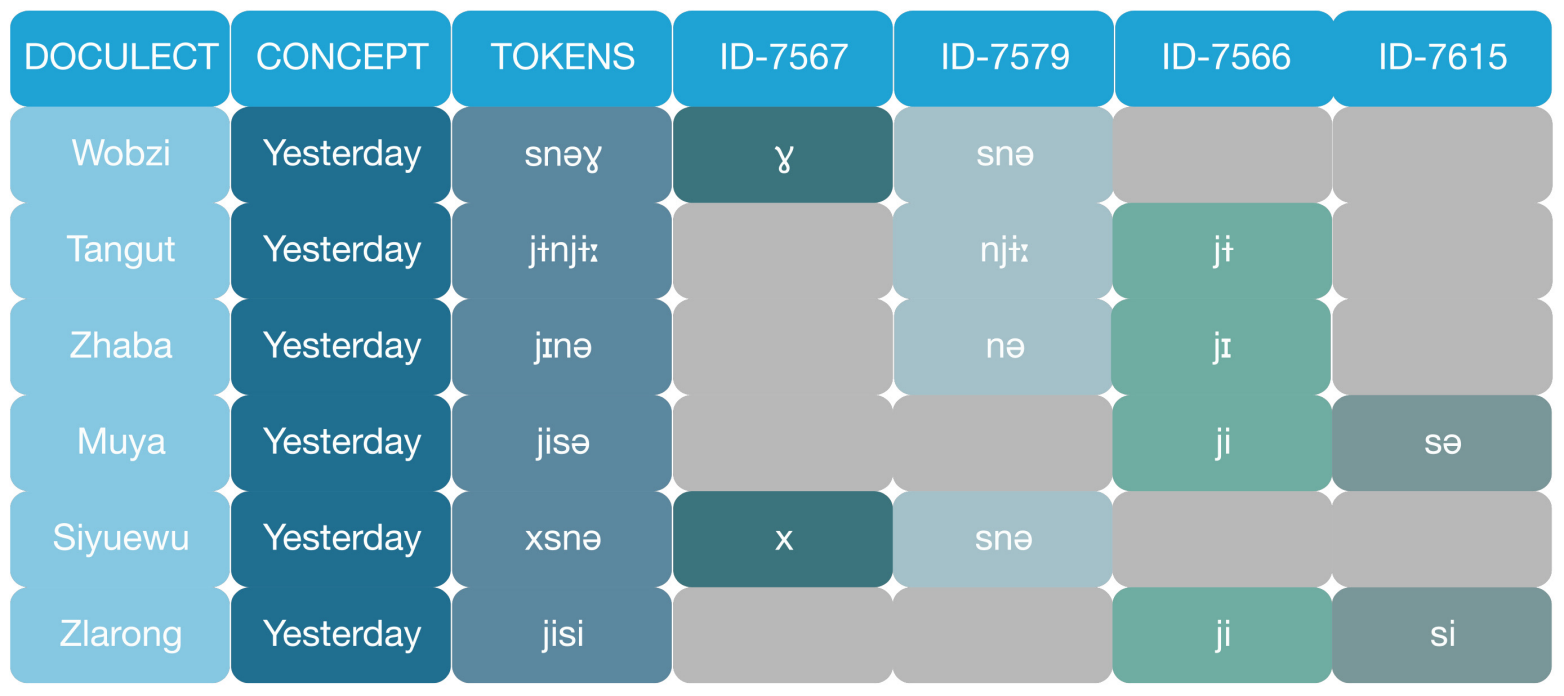
Partial cognate annotation: The concept ‘yesterday’ in Rgyalrongic is a compound of ‘past’ (cognates ID-7566 or ID-7567) and ‘day’ (cognates ID-7579 or ID-7615). Languages differ in the choices of the two parts. Annotating the two parts separately allows us to visualise and analyse the internal morphology of the forms for more accurate inference of phylogeny.

Using an orthography profile, the transcriptions were converted according to a unified standard for potential reuses. Phonemic and morphemic segmentation, as well as cognate judgements are carefully processed with expert knowledge. See Figure 3.

## Discussion and conclusion

Rgyalrongic languages are one of the most essential keys to the reconstruction of Proto-Sino-Tibetan as well as to the subgrouping of this language family. Although there exist searchable databases such as STEDT ^32^ ( https://stedt.berkeley.edu/) and the rGyalrongic Language Database ^33^ ( https://htq.minpaku.ac.jp/databases/rGyalrong/), the present database is the first Rgyalrongic lexical database that involves expert data curation and cognate annotation, and the only one that is ready for phylogenetic analyses.

For now, only those morphemes are assigned to the same cognate set which occur in words sharing the same meaning. In the future, we hope to extend this analysis to account for cognates across meaning slots, specifically concentrating also on language-internal partial cognates along the lines of the analysis pioneered in Hill and List ^21^ and further extended in Schweikhard and List ^34^. Having annotated the data in this form, cognacy can also be annotated on the word level ^35^ and computational approaches to phylogenetic reconstruction of Rgyalrongic (and beyond) can be carried out. Thus, the present contribution may serve as the very base of future phylogeny of one of the most conservative sub-branches of Sino-Tibetan.

## Data Availability

Data and Software available from: https://github.com/lexibank/lairgyalrong/. Archived source code and data at time of publication: https://doi.org/10.5281/zenodo.7866796 License: Creative Commons Attribution 4.0 International license (CC-BY 4.0)
