# Supplementary material for: Lexical data for the historical comparison of Rgyalrongic languages
Source: Open Res Eur. 2023 Oct 18;3:99. Originally published 2023 Jun 20. [Version 2] doi: 10.12688/openreseurope.16017.2 (PMC10446046; doi:10.12688/openreseurope.16017.2)
Supplement: Peer review history

# Reviewer report: Reviewer response for version 2

**DOI:** 10.21956/openreseurope.18034.r35685

**Published:** 2023-10-30

**Authors:** Mark Alves

**Affiliations:** 1 Montgomery College, Rockville, Maryland, USA

I have read over the authors' amended version and checked with my original comments. It seems suitable to me at this point for "Approved" status.

Are sufficient details of methods and materials provided to allow replication by others?

Partly

Is the rationale for creating the dataset(s) clearly described?

Yes

Are the datasets clearly presented in a useable and accessible format?

Yes

Are the protocols appropriate and is the work technically sound?

Yes

Reviewer Expertise:

NA

I confirm that I have read this submission and believe that I have an appropriate level of expertise to confirm that it is of an acceptable scientific standard.

# Reviewer report: Reviewer response for version 2

**DOI:** 10.21956/openreseurope.18034.r35683

**Published:** 2023-10-18

**Authors:** Alexis Michaud

**Affiliations:** 1 LACITO - Langues et civilisations à tradition orale, Paris, France

Version 2 addresses the concerns brought up, making good progress in terms of clarity of the online data set.

Are sufficient details of methods and materials provided to allow replication by others?

Partly

Is the rationale for creating the dataset(s) clearly described?

Yes

Are the datasets clearly presented in a useable and accessible format?

Yes

Are the protocols appropriate and is the work technically sound?

Yes

Reviewer Expertise:

NA

I confirm that I have read this submission and believe that I have an appropriate level of expertise to confirm that it is of an acceptable scientific standard.

# Reviewer report: Reviewer response for version 1

**DOI:** 10.21956/openreseurope.17295.r33646

**Published:** 2023-07-31

**Authors:** Norihiko Hayashi

**Affiliations:** 1 Kobe City University of Foreign Studies, Kobe, Japan

First of all, I would like to express my excitement over the recent release of a new database for the rGyalrongic (‘Rgyalrongic’ spelled in this paper) languages. However, I must clarify that I am not an expert in the rGyalrongic languages; rather, my specialization lies in Lolo-Burmese languages. Moreover, I have limited familiarity with statistical methods. Therefore, please be kindly notified that my review may incorporate some perspectives from classical historical linguistics.

 As stated in the paper, this database is a compilation of lexical data from nearly 20 languages classified as rGyalrongic. The authors themselves conducted research on two of the languages, while the remaining data either underwent careful analysis by experts in the respective languages or were extracted from dictionaries with detailed word annotations. The finalized database has been published on their website with the cooperation of these experts.

 The rGyalrongic languages, as the authors point out, exhibit an exceptionally morphologically complex lexicons among the Tibeto-Burman languages. This complexity not only makes it challenging to explore their historical development but also hinders the investigation of historical relationships among these languages. Until now, historical linguists familiar with these languages have relied on comparative linguistics methods and their own specialized judgment to classify the historical development and phylogeny based on morphological analysis of each language's vocabulary. However, this approach naturally has its limitations.

 This paper introduces a system that mitigates assumptions about the historical development of lexicons in each language by incorporating new technologies, search techniques, and even statistical methods. It unveils new possibilities for comparative studies. To achieve this, the paper aims to create a database with maximum scientific transparency, relying on a scrupulous selection of data sets and reliable information provided by experts proficient in each language.

 While it has its merits, it also has its drawbacks. To begin with, the attempt is still in its infancy in terms of its overall goal, so one can only hope for further development. The data for rGyalrongic languages is still very limitedly employed. Of course, I comprehend that the principal objective of this project is to meticulously curate and present data in collaboration with experts. However, it is still important to increase the amount of data regarding the future. The author himself mentions that a database of the rGyalrongic language, supervised by Nagano & Prins (2013), is available on the website, where the basic vocabulary and examples of sentences of 81 rGyalrongic languages are uploaded with their sound files. Indeed, similar to the STEDT database, it is essentially a collection of data lacking morphological analysis. However, in the future, with the cooperation of the supervisors, it would be possible to use the data from Nagano & Prins (2013) to conduct a more precise analysis of historical change.

 It should be noted that five of the languages in the dataset (Daofu, Zhaba, MaerkangrGyalrong, MuyaKangding, and QueyuXinlong) are taken from Huang (1992). Huang (1992) was a groundbreaking work at the time, a comparative lexicon of primary sources of Tibeto-Burman languages in China, which is very useful for historical and comparative studies. Unfortunately, Huang (1992) contains many printing errors, and the researcher, Prof. Huang Bufan, is deceased, thus we cannot ask her about her research. There are researchers, such as Satoko Shirai and Huang Yang on Zhaba (Gong 2007 is also a reference grammar of Zhaba), and Takumi Ikeda on Muya. For Situ rGyalrong, Yasuhiko Nagano also recently published a grammar in English (Nagano 2022). With the cooperation of these researchers who are still active in the field, there should be room for additional data and comparative studies in the future.

 The paper presents the partial cognate annotation by exemplifying a word ‘yesterday’ in Figure 3, which this paper considers is a compound of ‘day’ and ‘past’. This figure leads us to think that /ɣ/ in the Wobzi word /snə **ɣ**/ corresponds to /x/ in the Siyuewu word / **x**snə/ and both /ɣ/ and /x/ denote the concept ‘past’. This is really inspiring, and if we search the same word in Nagano & Prins 2013 database, there are many cases similar to {pə/ ma/ wa}+{snə}, like /pəʃe’ʃni/ (Jinchuan Jimu Zhouchan), /ma sȵi lo/ (Maerkang Soman), /mbasni’lo/ (Hongyuan Shuajinsi Selong), /wə ^3^ʂo ^3^ʃnə ^5^/ (Jinchuan Manai Genza), etc. The initial syllable of these words might indicate 'past,' which should be noted in the Rangtang Puxi Siyaowucun word / **ɣ**snə/, having the same meaning as 'yesterday'.

 Additionally, Nagano (2022) also provides a few intriguing expressions for ‘yesterday’, warranting a comparative analysis. Collaborating with experts and integrating data from other databases could significantly enhance the development of this project.

 Regarding the dataset of this project, while Bantawa and Old Burmese, languages that are quite distant historically from rGyalrongic languages, are included, Written Tibetan and Amdo Tibetan are not included in this dataset. Of course, this may be because there are many cases of Tibetan languages providing loanwords to rGyalrongic, but this situation may require some explanation. As for Old Burmese, it certainly has a phonetic writing system and the date of use is clear, so it is possible that the historical relationship between the two languages can be put on an absolute time scale. However, if the Bantawa data are to be combined, it seems to me that more Qiangic languages should be introduced, including Pumi and various Qiang dialects.

 At any rate, as mentioned earlier, this database will continue to develop, and it is expected that the data will be updated in the future. At the moment, this database still needs more user-friendly interfaces which are already presented as in the STEDT and Nagano & Prince’s databases. However, at the same time, if researchers of Tibeto-Burman languages apply the methodology of this project and develop datasets for each subgroup, it will be possible to discover new problems of historical change and to solve existing problems. At the very least, if the Lolo-Burmese dataset, which is the reviewer's specialty, is developed in a similar manner, it will allow for a more scientific analysis of the relationships between languages within Lolo-Burmese (which may modify the framework of Matisoff 1972, 2003, and Bradley 1979). It will also lead to a more detailed elucidation of the relationship between rGyalrongic and Lolo-Burmese, which was discussed here, than has been the case so far. We are convinced that this project deserves further attention.

Are sufficient details of methods and materials provided to allow replication by others?

Yes

Is the rationale for creating the dataset(s) clearly described?

Yes

Are the datasets clearly presented in a useable and accessible format?

Yes

Are the protocols appropriate and is the work technically sound?

Yes

Reviewer Expertise:

My specialties are Tibeto-Burman Linguistics, especially Lolo-Burmese languages. I have also interests in documenting Tai-Kadai languages in Mainland Southeast Asia.

I confirm that I have read this submission and believe that I have an appropriate level of expertise to confirm that it is of an acceptable scientific standard.

## References

[1] : Proto-Loloish. *Malmö: Curzon Press* .1979;

[2] : Zhaba-yu yanjiu. [Study of the Zhaba Language. (in Chinese)]. *Beijing: Minzu chubanshe* .2007;

[3] Zangmian yuzu yuyan cihui. [A Tibeto-Burman Lexicon. (in Chinese)]. *Beijing: Zhongyang minzu xueyuan chubansh* .1992;

[4] : *Berkeley: University of California Press* .Handbook of Proto-Tibeto-Burman;

[5] : Handbook of Proto-Tibeto-Burman. *Berkeley: University of California Press* .2003;

[6] : rGyalrong: A Comprehensive Grammar. *Tokyo: Fukyosha* .2022;

[7] : The rGyalrongic Languages Database.2013; Reference source

[8] : Reference source

# Response

**Published:** 2023-10-18

**Authors:** Yunfan Lai

**Affiliations:** School of Linguistic, Speech a, The University of Dublin Trinity College, Dublin, Leinster, Ireland

- While it has its merits, it also has its drawbacks. To begin with, the attempt is still in its infancy in terms of its overall goal, so one can only hope for further development. The data for rGyalrongic languages is still very limitedly employed. Of course, I comprehend that the principal objective of this project is to meticulously curate and present data in collaboration with experts. However, it is still important to increase the amount of data regarding the future. The author himself mentions that a database of the rGyalrongic language, supervised by Nagano & Prins (2013), is available on the website, where the basic vocabulary and examples of sentences of 81 rGyalrongic languages are uploaded with their sound files. Indeed, similar to the STEDT database, it is essentially a collection of data lacking morphological analysis. However, in the future, with the cooperation of the supervisors, it would be possible to use the data from Nagano & Prins (2013) to conduct a more precise analysis of historical change.
  - We are gradually expanding the data size with recent fieldwork under Yunfan Lai’s project. Yunfan’s project is now including two new varieties, Yaoji and Bragsteng into the database. They will be added as soon as data curation is done. Yunfan is also supervising an undergraduate student who works on a new West Gyalrongic variety. At least three new varieties with first hand data will be added to the database shortly. It is indeed a good idea, as the reviewer suggests, to explore more the Nagano and Prins database to see how we can collaborate. 
- It should be noted that five of the languages in the dataset (Daofu, Zhaba, MaerkangrGyalrong, MuyaKangding, and QueyuXinlong) are taken from Huang (1992). Huang (1992) was a groundbreaking work at the time, a comparative lexicon of primary sources of Tibeto-Burman languages in China, which is very useful for historical and comparative studies. Unfortunately, Huang (1992) contains many printing errors, and the researcher, Prof. Huang Bufan, is deceased, thus we cannot ask her about her research. There are researchers, such as Satoko Shirai and Huang Yang on Zhaba (Gong 2007 is also a reference grammar of Zhaba), and Takumi Ikeda on Muya. For Situ rGyalrong, Yasuhiko Nagano also recently published a grammar in English (Nagano 2022). With the cooperation of these researchers who are still active in the field, there should be room for additional data and comparative studies in the future.
  - We are well aware that Huang’s data are not free from errors. We are going to replace a part of her data with new, recent data. Yes, we will ask Shirai Satoko and Huang Yang for Zhaba data. We will include Cogtse data investigated by Lin You-Jing and Bhola data by Nagano. 
- The paper presents the partial cognate annotation by exemplifying a word ‘yesterday’ in Figure 3, which this paper considers is a compound of ‘day’ and ‘past’. This figure leads us to think that /ɣ/ in the Wobzi word /snəɣ/ corresponds to /x/ in the Siyuewu word /xsnə/ and both /ɣ/ and /x/ denote the concept ‘past’. This is really inspiring, and if we search the same word in Nagano & Prins 2013 database, there are many cases similar to {pə/ ma/ wa}+{snə}, like /pəʃe’ʃni/ (Jinchuan Jimu Zhouchan), /ma sȵi lo/ (Maerkang Soman), /mbasni’lo/ (Hongyuan Shuajinsi Selong), /wə3ʂo3ʃnə5/ (Jinchuan Manai Genza), etc. The initial syllable of these words might indicate 'past,' which should be noted in the Rangtang Puxi Siyaowucun word /ɣsnə/, having the same meaning as 'yesterday'. Additionally, Nagano (2022) also provides a few intriguing expressions for ‘yesterday’, warranting a comparative analysis. Collaborating with experts and integrating data from other databases could significantly enhance the development of this project.
  - The reviewer’s comments are really interesting. In our recent fieldwork, we have cognates (such as pəʃesni) mentioned by the reviewer, so our new first hand data will certainly increase the diversity of the entire work. One thing to point out: Rangtang Siyaowucun (in Nagano and Prin’s database) is exactly Siyuewu in our database, and we are confident that our notation is much more accurate, because it is from Yunfan Lai’s fieldwork from 2014 onwards. 
- Regarding the dataset of this project, while Bantawa and Old Burmese, languages that are quite distant historically from rGyalrongic languages, are included, Written Tibetan and Amdo Tibetan are not included in this dataset. Of course, this may be because there are many cases of Tibetan languages providing loanwords to rGyalrongic, but this situation may require some explanation. As for Old Burmese, it certainly has a phonetic writing system and the date of use is clear, so it is possible that the historical relationship between the two languages can be put on an absolute time scale. However, if the Bantawa data are to be combined, it seems to me that more Qiangic languages should be introduced, including Pumi and various Qiang dialects.
  - For the purpose of phylogenetic inference, we need to select one or two obvious outgroups, which are known to be remotely related, or totally unrelated, to the branch under analysis. Many studies select only one, but here we have included two, Bantawa and Old Burmese. We did not select them randomly, but on purpose. Bantawa is remotely related to Rgyalrongic, which help us to identify the Rgyalrongic branch from a Sino-Tibetan/Trans-Himalayan perspective. Old Burmese are more closely related to Rgyalrongic, but not Rgyalrongic per se, therefore, it can let us know whether our Rgyalrongic is accurately computed without merging with Old Burmese. 
- At any rate, as mentioned earlier, this database will continue to develop, and it is expected that the data will be updated in the future. At the moment, this database still needs more user-friendly interfaces which are already presented as in the STEDT and Nagano & Prince’s databases. However, at the same time, if researchers of Tibeto-Burman languages apply the methodology of this project and develop datasets for each subgroup, it will be possible to discover new problems of historical change and to solve existing problems. At the very least, if the Lolo-Burmese dataset, which is the reviewer's specialty, is developed in a similar manner, it will allow for a more scientific analysis of the relationships between languages within Lolo-Burmese (which may modify the framework of Matisoff 1972, 2003, and Bradley 1979). It will also lead to a more detailed elucidation of the relationship between rGyalrongic and Lolo-Burmese, which was discussed here, than has been the case so far. We are convinced that this project deserves further attention.
  - As in the response to Reviewer 1, we are going to provide the Edictor access to readers and users, so that everybody can review our cognate annotation and identification. Future work will focus on making something like the database of Nagana and Prins (2011).

# Reviewer report: Reviewer response for version 1

**DOI:** 10.21956/openreseurope.17295.r33643

**Published:** 2023-07-27

**Authors:** Mark Alves

**Affiliations:** 1 Montgomery College, Rockville, Maryland, USA

SUMMARY: This data report presents the status of ongoing historical linguistic research on the Rgyalrongic language group within Sino-Tibetan. It has involved assembling lexical data, identifying cognates, and preparing the data to use in phylogenetic study. The data includes thousands of words from 20 languages (mostly modern language, but the long-dead Tangut language) from a variety of previous studies. The goal is to make this data available for others with similar goals to use and, potentially, re-evaluate/re-test.

 METHODOLOGY: The approach in assembling the data is proper: selection and organization of lexical data of as many doculects of Rgyalrongic as possible, identification of cognates / partial cognates, semantic and phonetic standardization (to a reasonable degree), and preparing in files useable with software that can do phylogenetic work.

 There is one methodological matter I think worth giving attention to. While the assessment of cognates certainly must be done by researchers with relevant training and experience in working with the languages, such that they have trustworthy intuition about cognate status (your repeated use of the word "expert" in the article, which I think should be reduced), it would also be useful to acknowledge that phonological patterns were part of your mental criteria. You cannot and should not cover Rgyalrongic historical phonology in this short report, but a sentence or two mentioning that your intuition as researchers familiar with the languages involved noting recurring patterns (e.g., instances of consistency, but patterns of differences in consonants or vowels, etc.), along with a reference of a couple key references addressing some of these patterns, would help clarify the cognate identification process beyond "trustworthy intuition".

 DATA AND SOFTWARE: While all the data is readily available, some guidance / tips on using the data would be helpful to some potential users.

 SUGGESTIONS

 Below is a list of suggestions for editing, wording, and clarity of details. Some are optional, some are necessary, and some are strongly recommended.

- Regarding "The present lexical data sets," consider "The lexical data sets the authors have assembled."
- "are collected...from 2011 to 2016" > "were collected...from 2011 to 2016"
- In "expertise in Rgyalrong historical linguistics through the neogrammarian comparative method", the phrase "through the neogrammarian comparative method" is unclear in relation to the preceding main clause. I suggest either "through application of the neogrammarian comparative method" or perhaps "expertise in research on Rgyalrongic historical linguistics through the neogrammarian comparative method" (I don't think "neogrammarian" is needed).
- segmantation > segmentation 
- "albeit including" doesn't connect to the previous sentence smoothly. Consider something like "though Tangut, an extinct medieval language, was spoken in Ningxia Province."
- "For most non-native speakers, they are the most difficult branch of languages to learn..." is odd. Non-native speakers of all Sino-Tibetan languages? Just remove "for most non-native speakers". 
- "...they exhibit a large array of word formation strategies such as inflection and derivation..." is not logical since for "a large array", you can only list inflectional and derivational morphology (but perhaps reduplication). I think you mean that the inflectional and derivational morphology has a complex range of morphophonological patterns, combined with complex morphosyntax.
- Instead of "old age," perhaps "historical depth in the language group".
- Instead of "finding out the origin of Sino-Tibetan", perhaps "exploring Sino-Tibetan language history."
- Regarding "This database aims...", since this is an article, not a database, you need to write something like "The database considered in this article" or something like that.
- Instead of "Rgyalrongic languages are a Sino-Tibetan branch, mainly spoken...", perhaps "Rgyalrongic languages form a Sino-Tibetan branch and are mainly spoken..."
- "They are closely related to Lolo-Burmese languages" is minimal information, especially for non-specialists. Consider "They belong to the Qiangic sub-branch of Burmo-Qiangic and are thus closely related to Lolo-Burmese languages."
- "...a medieval language, Tangut, is recently recognised to belong to said branch" > "...the extinct Tangut language has been recently recognized as a Rgyalrongic language" or "has been recently determined to belong to this sub-branch".
- "...languages, Situ, ..." > "...languages: Situ, ..."
- fuß > Fuß
- The sentence "Thus, it is very likely that language varieties closely linked to Zhaba, such as Queyu and Minyag (aka. Muya, Menya) and Zlarong spoken in the Tibetan **Autonmous (> Autonomous) **Region, are also Rgyalrongic languages" is confusing. Since you include these languages in the study, "it is very likely" sounds too tentative, so consider "we have considered....to be Rgyalrongic languages in our study".
- In the phrase "These “newcomers”...", "newcomers" is somewhat ambiguous. Perhaps "new additions to the Rgyalrongic group"?
- The statement "certainly the closest to Proto-Sino-Tibetan compared to all other varieties in China" is problematic. First, while there are many reconstructions of aspects of Sino-Tibetan morphology, these are not (to my knowledge) agreed upon to a major degree, which makes your claim premature. I agree that Rgyalrongic's complex morphological system suggest historical depth, and undoubtedly sheds light on Proto-Sino-Tibetan. But statements such as "certainly the closest...compared to all..." is a statement that would need substantial support to make, which is not the goal of this report. A statement such as "and it has a complex and, in our view, highly conservative morphological system that may give hints as to ancient features in the Sino-Tibetan language family", or instead of "may give", "has given" if you have a publication to cite with such support.
- "for the study on" > "for the study of"
- Instead of "The phylogeny of Rgyalrongic with dating information" ("with dating information" is awkward), consider "Phlogenetic research on Rgyalrongic to provide chronological information."
- For "has been proven to show accurate results3,5", consider "has been proven to show accurate results in both Sino-Tibetan and other language families3,5" since that is what the two references show.
- "high quality curation is inevitable" > "high quality curation is essential"
- "the phylogenetic analyses of Rgyalrongic languages" > "the phylogenetic analysis of Rgyalrongic languages"
- Instead of "It includes", consider "It contains lexical data from".
- Instead of "The entire workflow", consider "The workflow".
- Instead of "a specifically designed and curated," consider "a designed and curated".
- "has already been collected before 2017" < "was collected before 2017"
- "Dictionaries and word lists" > "These dictionaries and word lists"
- "Some word lists" > "Some of the word lists"
- Reorganize the following for standard presentation of items: "Reliability is assessed through two aspects: i) internal phonological consistency of the source data. The authors check if phonemes are correctly identified and that allophones conditioned by phonological environments and morphological alternations are adequately represented in the original sources. ii) external regularity of sound correspondences with the comparative method." > "Reliability is assessed through two aspects: i) internal phonological consistency of the source data and ii) external regularity of sound correspondences with the comparative method. The authors check if phonemes are correctly identified and that allophones conditioned by phonological environments and morphological alternations are adequately represented in the original sources. In addition,..."
- "The authors check if..." > "The authors checked if..." (Parallel to the past tense in the subsequent sentence)
- In "...minor differences. Queyuxinlong and QueyuPubarong", consider "minor differences. In contrast, Queyuxinlong and QueyuPubarong" for evident contrast emphasis.
- Be careful: "These two languages are remotely related to Rgyalrongic" of which one is Old  Burmese in Lolo-Burmese appears to contrast with your statement that Lolo-Burmese is closely related. You might need to replace "closely" in the previous instance since a time depth of 4,300 years suggests more linguistic distance, albeit within a common branch.
- "...sufficiently remote to..." > "...sufficiently remote from..." 
- Instead of "largely facilitates", consider "facilitates".
- Suggestion: ", however," > ". However," and ", therefore" > ". Therefore,"
- Instead of "which are however not widely distributed from the perspectives of the entire Sino-Tibetan family", consider "which are not widely distributed in other branches of Sino-Tibetan."
- "included ‘girl’, as" > included ‘girl’ as"
- "languages, we therefore" > "languages. We therefore"
- "consider them as worthwhile to be included in the dataset" > "consider them worth including in the dataset"
- "from each other, some may" > "from each other, and some may"
- "can be deducted" > "can be deduced"
- The sentence "For instance, Mandarin Chinese yuè-liang ‘moon’ and Taiwanese Southern Min guēh-niûn ‘moon’ only share the first part yuè and guēh, respectively, as cognates" is not ideal as Sinitic is typologically divergent, and this example of bisyllabic compounds doesn't--in my view--sufficiently highlight the challenges in cognacy identification in polysyllabic languages in other branches of Sino-Tibetan. It would be best to show an example of this complex situation from Sino-Tibetan languages in other branches, and that have affixation of different sources, but cognate roots, not compounding. However, if you do keep this example, it would help to clearly show that they share 月, but that liang is for 亮 while Taiwanese niu is for 娘 (with the kind of certainty than cannot be offered for languages outside of Sinitic), and are thus distinct etyma.
- The sentence "Partial cognates enable us to segment word forms into morphemes, which improves the accuracy of the computation of language subgrouping" is not quite right. First, the cognates don't allow segmentation: identification of partial cognates does this. Next, the cause of improved accuracy of computational subgrouping of the languages is the improved accuracy of cognate identification by marking both full and partial cognates. Consider rewording this.
- Question regarding "...annotate partial cognates, rather than full cognates...", does this mean there are no full cognates in the data? That may be possible if all words are morphologically marked with affixes. If so, make that clear. If not, "rather than" needs to be fixed.
- Perhaps there are different styles, but I generally use commas with numbers such as "6335" > "6,335" except when they are years. If this is standard for this publication, that's fine. Otherwise, please check throughout the paper.
- Regarding "...22 distinct language varieties", your abstract mentions 20. Please clarify.
- The phrase "413 distinct sounds" is ambiguous. I assume it mean "speech sounds" (not phonemes since that would require phonemic assessment). You might add "(i.e., consonants, vowels, and tones)" (if tones are among the sounds).
- "average inventory size of 72 different sounds" > Consider "average inventory of 72 distinct sounds". sounds"
- Regarding "The current dataset contains a total of 6335 word forms for 22 distinct language varieties. Word forms correspond to 305 different concepts," does this mean that 6,335 word forms fit into just 305 meanings? It's possible, but I would expect a larger number unless the wordlists are all short, as fieldwork lists sometimes are. Or do you mean that you focused on identification of words in 305 concepts? Please make this clear.
- Regarding "These morphemes have been assigned to 3109 cognate sets. Of these cognate sets, 1665 are unique, which means that we could not identify related words in other languages," this seems to say that 1,665 cognates sets do not have cognates in other languages, which is contradictory. Please rewrite to clarify.
- Regarding "We carefully verified the data to ensure the accuracy and correctness," the last part is general. First, "accuracy" and "correctness" are synonyms, so just use "accuracy", and then add briefly accuracy of what specifically about the data? That is was entered correctly from the sources, and/or other aspects? Clarifying this can increase confidence.
- I don't recommend overusing "expert, so for "We use our expert knowledge to review every lexical entry in the database" > "We reviewed every lexical entry in the database." However, reviewed them for what specifically? What kinds of problems were or aspects were you concerned with? You can just say "We checked the data," but that's uninformative.
- "potential reuses" > "potential reuse" (noncount noun)
- "judg **e**ments are" > "judgments, are"
- Again "...are carefully processed with expert knowledge", perhaps just "...were processed based on our experience in working with languages in the region" or something like that.
- Regarding "Languages differ in the choices of the two parts.", please clarify. Perhaps "The word forms differ in terms of cognacy of different morphemes"???
- Regarding "...for more accurate inference of phylogeny," this is data that is computationally analyzed, so not inferenced, right? Perhaps "...for more accurate data used for computational phylogenetic analysis."
- Regarding "Rgyalrongic languages are one of the most essential keys to the reconstruction of Proto-Sino-Tibetan as well as to the subgrouping of this language family," you must have at least a couple of references for this claim. If so, add them. If not, you need to hedge this statement, such as "We believe the Rgyalrongic languages have great potential value in reconstruction...".
- Again, "involves expert data curation and cognate annotation", do you need the word "expert"? One use in the article is sufficient, and this sentence is already clear without it.
- "phylogenetic analyses" > "phylogenetic analyses" (The noncount meaning is best here)
- Regarding "For now, only those morphemes are assigned to the same cognate set which occur in words sharing the same meaning.", this is confusing. Perhaps "For now, only those morphemes assigned to the same cognate set occur in words sharing the same meaning." Is that what you mean?
- Regarding "...to account for cognates across meaning slots", by "across meaning slots," do you mean near synonyms and/or instances of semantic extension, or something else? Please clarify.
- "concentrating also on" > "concentrating on" (Is "also" needed"?)
- "on the word level" > "at the word level"

MY RESEARCH BACKGROUND

 My research focus is on Austroasiatic, with Vietnamese as the center of this, but study of Vietnamese language history in particular requires much information about Chinese and Kra-Dai language history, and Southeast Asian language history broadly, all part of my broader research agenda. This involves sifting lexical data in digital databases for etymological study, naturally including cognate identification and interdisciplinary evidence of language contact. Also in Austroasiatic historical linguistics, I must deal with neighboring Tibeto-Burman languages and frequently consult STEDT (the Sino-Tibetan Etymological Dictionary)). However, while I have seen studies of Rgyalrongic languages, I have little specific knowledge of of them, though as I am interested in the ongoing research into Sino-Tibetan (aka Trans-Himalayan) language history, this brief report is of interest, both in terms of the potential use in Sino-Tibetan historical linguistics and in the methodology the authors used and challenges they face in identifying cognates.

Are sufficient details of methods and materials provided to allow replication by others?

Partly

Is the rationale for creating the dataset(s) clearly described?

Yes

Are the datasets clearly presented in a useable and accessible format?

Yes

Are the protocols appropriate and is the work technically sound?

Yes

Reviewer Expertise:

Historical linguistics (phonology, syntax, etymology, dialectology, language contact, inter-disciplinary approaches) of Vietnamese, Austroasiatic, and Southeast Asia broadly

I confirm that I have read this submission and believe that I have an appropriate level of expertise to confirm that it is of an acceptable scientific standard, however I have significant reservations, as outlined above.

# Response

**Published:** 2023-07-27

**Authors:** Yunfan Lai

**Affiliations:** School of Linguistic, Speech a, The University of Dublin Trinity College, Dublin, Leinster, Ireland

Dear Mark, thank you very much for your comments. They are very helpful indeed. We are still waiting for the third and probably final review, then we will do our best to revise the paper. Best, Yunfan

# Response

**Published:** 2023-10-18

**Authors:** Yunfan Lai

**Affiliations:** School of Linguistic, Speech a, The University of Dublin Trinity College, Dublin, Leinster, Ireland

- There is one methodological matter I think worth giving attention to. While the assessment of cognates certainly must be done by researchers with relevant training and experience in working with the languages, such that they have trustworthy intuition about cognate status (your repeated use of the word "expert" in the article, which I think should be reduced), it would also be useful to acknowledge that phonological patterns were part of your mental criteria. You cannot and should not cover Rgyalrongic historical phonology in this short report, but a sentence or two mentioning that your intuition as researchers familiar with the languages involved noting recurring patterns (e.g., instances of consistency, but patterns of differences in consonants or vowels, etc.), along with a reference of a couple key references addressing some of these patterns, would help clarify the cognate identification process beyond "trustworthy intuition".
  - This is a very good suggestion. In the first place, we have reduced the word “expert”. In the second place, we have mentioned some criteria, including “trustworthy intuition” or “educated guess” in the revised version. Recently, I have several new publications discussing sound correspondences among Rgyalrongic languages, I have also mentioned them when revising the paper. 

 

- Regarding "The present lexical data sets," consider "The lexical data sets the authors have assembled."
  - I have changed the wording accordingly. 

 

- "are collected...from 2011 to 2016" > "were collected...from 2011 to 2016"
  - I have changed the wording accordingly.

 

- In "expertise in Rgyalrong historical linguistics through the neogrammarian comparative method", the phrase "through the neogrammarian comparative method" is unclear in relation to the preceding main clause. I suggest either "through application of the neogrammarian comparative method" or perhaps "expertise in research on Rgyalrongic historical linguistics through the neogrammarian comparative method" (I don't think "neogrammarian" is needed).
  - I have changed the wording accordingly.

 

- segmantation > segmentation 
  - I have changed the wording accordingly.

 

- "albeit including" doesn't connect to the previous sentence smoothly. Consider something like "though Tangut, an extinct medieval language, was spoken in Ningxia Province."
  - I have changed the wording accordingly.

 

- "For most non-native speakers, they are the most difficult branch of languages to learn..." is odd. Non-native speakers of all Sino-Tibetan languages? Just remove "for most non-native speakers". 
  - I have changed the wording accordingly.

 

- "...they exhibit a large array of word formation strategies such as inflection and derivation..." is not logical since for "a large array", you can only list inflectional and derivational morphology (but perhaps reduplication). I think you mean that the inflectional and derivational morphology has a complex range of morphophonological patterns, combined with complex morphosyntax.
  - The reviewer is right about this point. I have selected a better wording. 

 

- Instead of "old age," perhaps "historical depth in the language group".
  - I have changed the wording accordingly.

 

- Instead of "finding out the origin of Sino-Tibetan", perhaps "exploring Sino-Tibetan language history."
  - I have changed the wording accordingly.

 

- Regarding "This database aims...", since this is an article, not a database, you need to write something like "The database considered in this article" or something like that.
  - I have changed the wording accordingly.

 

- Instead of "Rgyalrongic languages are a Sino-Tibetan branch, mainly spoken...", perhaps "Rgyalrongic languages form a Sino-Tibetan branch and are mainly spoken..."
  - I have changed the wording accordingly.

 

- "They are closely related to Lolo-Burmese languages" is minimal information, especially for non-specialists. Consider "They belong to the Qiangic sub-branch of Burmo-Qiangic and are thus closely related to Lolo-Burmese languages."
  - I have changed the wording accordingly.

 

- "...a medieval language, Tangut, is recently recognised to belong to said branch" > "...the extinct Tangut language has been recently recognized as a Rgyalrongic language" or "has been recently determined to belong to this sub-branch".
  - I have changed the wording accordingly.

 

- "...languages, Situ, ..." > "...languages: Situ, ..."
  - I have changed the wording accordingly.

 

- fuß > Fuß
  - I have also noticed this error. I have corrected it. 

 

- The sentence "Thus, it is very likely that language varieties closely linked to Zhaba, such as Queyu and Minyag (aka. Muya, Menya) and Zlarong spoken in the Tibetan Autonmous (> Autonomous) Region, are also Rgyalrongic languages" is confusing. Since you include these languages in the study, "it is very likely" sounds too tentative, so consider "we have considered....to be Rgyalrongic languages in our study".
  - We fully agree with the reviewer. 

 

- In the phrase "These “newcomers”...", "newcomers" is somewhat ambiguous. Perhaps "new additions to the Rgyalrongic group"?
  - We also prefer the suggestion of the reviewer. 

 

- The statement "certainly the closest to Proto-Sino-Tibetan compared to all other varieties in China" is problematic. First, while there are many reconstructions of aspects of Sino-Tibetan morphology, these are not (to my knowledge) agreed upon to a major degree, which makes your claim premature. I agree that Rgyalrongic's complex morphological system suggest historical depth, and undoubtedly sheds light on Proto-Sino-Tibetan. But statements such as "certainly the closest...compared to all..." is a statement that would need substantial support to make, which is not the goal of this report. A statement such as "and it has a complex and, in our view, highly conservative morphological system that may give hints as to ancient features in the Sino-Tibetan language family", or instead of "may give", "has given" if you have a publication to cite with such support.
  - The reviewer’s doubt is reasonable. Our claim is indeed premature. We have revised this passage in order to make it more scientifically viable. 

 

- "for the study on" > "for the study of"
  - I have changed the wording accordingly.

 

- Instead of "The phylogeny of Rgyalrongic with dating information" ("with dating information" is awkward), consider "Phlogenetic research on Rgyalrongic to provide chronological information."
  - I doubt that “chronological” is the appropriate term. Bayesian phylogeny gives exact estimations of dates, instead of chronology, of sub-branches. I have made this more comprehensible. 

 

- For "has been proven to show accurate results3,5", consider "has been proven to show accurate results in both Sino-Tibetan and other language families3,5" since that is what the two references show.
  - I have changed the wording accordingly.

 

- "high quality curation is inevitable" > "high quality curation is essential"
  - I have changed the wording accordingly.

 

- "the phylogenetic analyses of Rgyalrongic languages" > "the phylogenetic analysis of Rgyalrongic languages"
  - I have changed the wording accordingly.

 

- Instead of "It includes", consider "It contains lexical data from".
  - I have changed the wording accordingly.

 

- Instead of "The entire workflow", consider "The workflow".
  - I have changed the wording accordingly.

 

- Instead of "a specifically designed and curated," consider "a designed and curated".
  - I have changed the wording accordingly.

 

- "has already been collected before 2017" < "was collected before 2017"
  - I have changed the wording accordingly.

 

- "Dictionaries and word lists" > "These dictionaries and word lists"
  - I have changed the wording accordingly.

 

- "Some word lists" > "Some of the word lists"
  - I have changed the wording accordingly.

 

- Reorganize the following for standard presentation of items: "Reliability is assessed through two aspects: i) internal phonological consistency of the source data. The authors check if phonemes are correctly identified and that allophones conditioned by phonological environments and morphological alternations are adequately represented in the original sources. ii) external regularity of sound correspondences with the comparative method." > "Reliability is assessed through two aspects: i) internal phonological consistency of the source data and ii) external regularity of sound correspondences with the comparative method. The authors check if phonemes are correctly identified and that allophones conditioned by phonological environments and morphological alternations are adequately represented in the original sources. In addition,..."
  - I have changed the wording accordingly.

 

- "The authors check if..." > "The authors checked if..." (Parallel to the past tense in the subsequent sentence)
  - I have changed the wording accordingly.

 

- In "...minor differences. Queyuxinlong and QueyuPubarong", consider "minor differences. In contrast, Queyuxinlong and QueyuPubarong" for evident contrast emphasis.
  - I have changed the wording accordingly.

 

- Be careful: "These two languages are remotely related to Rgyalrongic" of which one is Old  Burmese in Lolo-Burmese appears to contrast with your statement that Lolo-Burmese is closely related. You might need to replace "closely" in the previous instance since a time depth of 4,300 years suggests more linguistic distance, albeit within a common branch.
  - Yes, this is a problem. I have fixed this.

 

- "...sufficiently remote to..." > "...sufficiently remote from..." 
  - I have changed the wording accordingly.

 

- Instead of "largely facilitates", consider "facilitates".
  - I have changed the wording accordingly.

 

- Suggestion: ", however," > ". However," and ", therefore" > ". Therefore,"
  - I have changed the wording accordingly.

 

- Instead of "which are however not widely distributed from the perspectives of the entire Sino-Tibetan family", consider "which are not widely distributed in other branches of Sino-Tibetan."
  - I have changed the wording accordingly.

 

- "included ‘girl’, as" > included ‘girl’ as"
  - I have changed the wording accordingly.

 

- "languages, we therefore" > "languages. We therefore"
  - I have changed the wording accordingly.

 

- "consider them as worthwhile to be included in the dataset" > "consider them worth including in the dataset"
  - I have changed the wording accordingly.

 

- "from each other, some may" > "from each other, and some may"
  - I have changed the wording accordingly.

 

- "can be deducted" > "can be deduced"
  - I have changed the wording accordingly.

 

- The sentence "For instance, Mandarin Chinese yuè-liang ‘moon’ and Taiwanese Southern Min guēh-niûn ‘moon’ only share the first part yuè and guēh, respectively, as cognates" is not ideal as Sinitic is typologically divergent, and this example of bisyllabic compounds doesn't--in my view--sufficiently highlight the challenges in cognacy identification in polysyllabic languages in other branches of Sino-Tibetan. It would be best to show an example of this complex situation from Sino-Tibetan languages in other branches, and that have affixation of different sources, but cognate roots, not compounding. However, if you do keep this example, it would help to clearly show that they share 月, but that liang is for 亮 while Taiwanese niu is for 娘 (with the kind of certainty than cannot be offered for languages outside of Sinitic), and are thus distinct etyma.
  - I have removed this example and replaced it with the example for yesterday in Figure 3, which is also in our database. 

 

- The sentence "Partial cognates enable us to segment word forms into morphemes, which improves the accuracy of the computation of language subgrouping" is not quite right. First, the cognates don't allow segmentation: identification of partial cognates does this. Next, the cause of improved accuracy of computational subgrouping of the languages is the improved accuracy of cognate identification by marking both full and partial cognates. Consider rewording this.
  - Yes. The emphasis on partial cognates is that it is the recent innovative technique developped by our team. I have reworded this to make it more accurate. 

 

- Question regarding "...annotate partial cognates, rather than full cognates...", does this mean there are no full cognates in the data? That may be possible if all words are morphologically marked with affixes. If so, make that clear. If not, "rather than" needs to be fixed.
  - There are indeed full cognates in this data. I have fixed this point. 

 

- Perhaps there are different styles, but I generally use commas with numbers such as "6335" > "6,335" except when they are years. If this is standard for this publication, that's fine. Otherwise, please check throughout the paper.
  - I have corrected it. 

 

- Regarding "...22 distinct language varieties", your abstract mentions 20. Please clarify.
  - There are two out-group languages, Bantawa and Old Burmese, apart from the 20 Rgyalrongic languages. I have clarified this point. 

 

- The phrase "413 distinct sounds" is ambiguous. I assume it mean "speech sounds" (not phonemes since that would require phonemic assessment). You might add "(i.e., consonants, vowels, and tones)" (if tones are among the sounds).
  - Yes. I have added them in the revised version. 
- "average inventory size of 72 different sounds" > Consider "average inventory of 72 distinct sounds". sounds"
  - I have corrected this. 

 

- Regarding "The current dataset contains a total of 6335 word forms for 22 distinct language varieties. Word forms correspond to 305 different concepts," does this mean that 6,335 word forms fit into just 305 meanings? It's possible, but I would expect a larger number unless the wordlists are all short, as fieldwork lists sometimes are. Or do you mean that you focused on identification of words in 305 concepts? Please make this clear.
  - The 6,335 word forms fit into the 305 concepts (meanings) in 22 languages, which I think is rather a reasonable number and distribution, since we cannot guarantee that every language has a word form for each of the 305 concepts. I have made this point more explicit in the revised version. 

 

- Regarding "These morphemes have been assigned to 3109 cognate sets. Of these cognate sets, 1665 are unique, which means that we could not identify related words in other languages," this seems to say that 1,665 cognates sets do not have cognates in other languages, which is contradictory. Please rewrite to clarify.
  - This is related to our definition of cognate sets. It is possible that a cognate set contains only one form from one language. In this case, this cognate set is unique. Unique cognate sets can be due to innovations of individual languages, or rare retentions from the proto-language. 
- Regarding "We carefully verified the data to ensure the accuracy and correctness," the last part is general. First, "accuracy" and "correctness" are synonyms, so just use "accuracy", and then add briefly accuracy of what specifically about the data? That is was entered correctly from the sources, and/or other aspects? Clarifying this can increase confidence.
  - I agree that this point needs more clarifications. I have added a passage on this issue. 

 

- I don't recommend overusing "expert, so for "We use our expert knowledge to review every lexical entry in the database" > "We reviewed every lexical entry in the database." However, reviewed them for what specifically? What kinds of problems were or aspects were you concerned with? You can just say "We checked the data," but that's uninformative.
  - I have changed the wording accordingly. 

 

- "potential reuses" > "potential reuse" (noncount noun)
  - I have corrected this. 

 

- "judgements are" > "judgments, are"
  - I have corrected this. 

 

- Again "...are carefully processed with expert knowledge", perhaps just "...were processed based on our experience in working with languages in the region" or something like that.
  - I have changed the wording with more information. 

 

- Regarding "Languages differ in the choices of the two parts.", please clarify. Perhaps "The word forms differ in terms of cognacy of different morphemes"???
  - Yes. This is not clear enough. I have changed the wording. 

 

- Regarding "...for more accurate inference of phylogeny," this is data that is computationally analyzed, so not inferenced, right? Perhaps "...for more accurate data used for computational phylogenetic analysis."
  - The reviewer is right about this point. I have revised the paper accordingly. 

 

- Regarding "Rgyalrongic languages are one of the most essential keys to the reconstruction of Proto-Sino-Tibetan as well as to the subgrouping of this language family," you must have at least a couple of references for this claim. If so, add them. If not, you need to hedge this statement, such as "We believe the Rgyalrongic languages have great potential value in reconstruction...".
  - I have added a couple of references. 

 

- Again, "involves expert data curation and cognate annotation", do you need the word "expert"? One use in the article is sufficient, and this sentence is already clear without it.
  - The reviewer is indeed right to point out my overuse of “expert”. 

 

- "phylogenetic analyses" > "phylogenetic analyses" (The noncount meaning is best here)
  - I have corrected the word. 

 

- Regarding "For now, only those morphemes are assigned to the same cognate set which occur in words sharing the same meaning.", this is confusing. Perhaps "For now, only those morphemes assigned to the same cognate set occur in words sharing the same meaning." Is that what you mean?
  - Yes, this is exactly what I mean. 

 

- Regarding "...to account for cognates across meaning slots", by "across meaning slots," do you mean near synonyms and/or instances of semantic extension, or something else? Please clarify.
  - I mean cognates with different meanings: for instance, sqí in Khroskyabs means ‘female siblings’, its cognate in Bragbar, ɕkʰɐ́j, means ‘female friend’. We will develop a workflow to deal with this kind of cross-semantic cognates. 

 

- "concentrating also on" > "concentrating on" (Is "also" needed"?)
  - I have corrected this. 
- "on the word level" > "at the word level"
  - I have corrected this. 

# Reviewer report: Reviewer response for version 1

**DOI:** 10.21956/openreseurope.17295.r32973

**Published:** 2023-07-07

**Authors:** Alexis Michaud

**Affiliations:** 1 LACITO - Langues et civilisations à tradition orale, Paris, France

**Summary of the submission:**

 This database gathers lexical information of Rgyalrongic languages to facilitate future research on historical and evolutionary linguistics. It includes word lists from fieldwork and published sources, providing data for phylogenetic analysis of the Sino-Tibetan family.

 An important disclaimer is that this reviewer has no first-hand knowledge of any Rgyalrongic language, and only contributes to historical-comparative work as part of a team that includes professional historical linguists. I guess that it is relevant to further clarify that my main research focus is on the Naish subgroup of Sino-Tibetan (Trans-Himalayan) – the Naxi, Na (Mosuo) and Laze languages –, and that the topic of Rgyalrongic reconstruction is of great interest to me, as progress in Rgyalrongic historical linguistics provides a gradually improved basis for establishing cognate sets between Rgyalrongic and Naish.

 The data note «Lexical data for the historical comparison of Rgyalrongic languages» introduces the current version of a database which, as can be looked up from the stats of the GitHub repository, has been set up in early 2020 and has undergone significant reworking in the Spring of 2022. Publication on the Open Research Europe platform will hopefully help clarify to a wide audience the usefulness of the database in its present state, as well as draw attention to a number of forward-looking features in its architecture. The database is a cutting-edge research tool for the comparative study of Rgyalrongic in its Sino-Tibetan context. The database does not aim to aggregate *lexical data* for historical-linguistic purposes, as done e.g. in the Sino-Tibetan Etymological Dictionary and Thesaurus (STEDT) project at the University of California at Berkeley. The database under review only contains 305 hand-picked cognate sets, currently amounting to a total of 6,335 word forms (for 22 distinct language varieties). By contrast, the STEDT lexical file is more than an order of magnitude larger, containing over 376,000 words in about 200 languages and dialects. Looking for the word ‘nose’ in STEDT ( https://stedt.berkeley.edu/~stedt-cgi/rootcanal.pl/gnis?t=nose), one gets a set of no less than 1,241 records, with twelve separate etyma. The entries are arranged by language subgroups, without cognacy judgments. There are 129 entries for six Rgyalrongic languages. These languages are (adding, in brackets, the corresponding names in the work under review): Ganzi Danba Geshenzha (Geshizha), Daofu/Ergong (Daofu/Mazur), Lavrung (Khroskyabs), Minyag (Muya/Menya), and several dialects of Rgyalrong. The database under review is much smaller in terms of number of items (again, by nearly an order of magnitude), but has better language coverage (covering about twice as many languages) and, crucially, only returns one item for the intended word: the cognate decided upon manually by the expert in charge of the database (the corresponding author of the data note, LAI Yunfan). There are thus fundamental differences between the STEDT database and the database under review. The STEDT, complete with an online user interface, is a functional and versatile large-scale tool that offers ample opportunity for ‘data crunching’, including peeking at items that are semantically related (‘to blow one’s nose’, ‘nose flaps’...). By contrast, the database under review is a diamond point: it is small by design, intended as a building-block for state-of-the-art explorations in computer-assisted historical linguistics. Use of standardized formats allows it to be plugged into a range of computational pipelines, to be used in association with other datasets as required for a specific research purpose.

 From the point of view of research methods, the database under review is up to the highest standards from several perspectives: that of Open Science (the data note itself is published under a permissive CC BY license), that of Sino-Tibetan historical linguistics (as the data curation process is exemplary), and that of interdisciplinary work associating linguistics and computer science. Such lovingly handcrafted, computation-friendly cognate sets allow for a seamless integration of the time-honoured methods of historical linguistics with computational approaches. It is to be hoped that other datasets that are key to progress in Sino-Tibetan historical linguistics, such as Old Chinese reconstructions in the Baxter-Sagart system (currently hosted at a custom website, and thus not looking very ‘future-proof’: https://ocbaxtersagart.lsait.lsa.umich.edu/), will find their way to (i) archives ensuring long-term conservation and (ii) hubs routinely used by computational linguists and computer scientists, such as GitHub, GitLab, Bitbucket and such – the landscape here changes rapidly, but the investment of publishing datasets on these platforms is well worth the effort, as it greatly facilitates interdisciplinary collaborations.

 On a slightly critical note, the data note presenting the database does not appear to me to do full justice to the database’s design and applications. In the «Plain language summary», the authors’ description of the database as work «in preparation for future work on historical and evolutionary linguistics» strikes me as unnecessarily modest. Perspectives of uses of the database are pushed back into a somewhat vague future, as if the database were intended as food for thought for linguists living at some point along the (theoretical) line of digital eternity. True, posterity may be grateful and appreciative, but the same very general point could be made about any other data set that gets curated and archived. In real life, not that many of the datasets in digital archives such as Zenodo may eventually be used in future, and still fewer may prove useful in research. By contrast, the database of rGyalrongic languages is not only a functional tool for research: it could be argued to be, by now, a tool whose usefulness has been tried and tested. The tool was being used at the same time as it was being set up. In computational terms, one could say that public release of the database is a transition from active ‘alpha-testing’ to ‘beta-testing’ by an open-ended list of end-users. Moreover, new end-users could potentially also serve as contributors in the mid run, for other cognate sets that may include a different choice of languages and/or concepts. The authors of the database are part of the team of authors of an influential 2019 article arguing that «Dated language phylogenies shed light on the ancestry of Sino-Tibetan»; there are clear shared features between the cognate sets used in the 2019 article and in the database under review, such as use of the second author’s Concepticon. Thus, the database under review is part of a growing body of data and tools that possesses demonstrated value to the field of Sino-Tibetan diachronic research. I would therefore suggest rephrasing the relevant passage of the «Plain language summary» from «in preparation for future work on historical and evolutionary linguistics» to «as a tool for research in the field of historical and evolutionary linguistics».

 Concerning matters of form, the data note could do with an additional round of text editing to ensure best readability for authors outside the first circle of specialists of this area of Sino-Tibetan. There are 20 languages in Figure 1, and 22 in Table 1. It would be useful to provide clarifications in the caption to Figure 1. Quick fix: *Figure 1. Geographical distribution of the* ***Rgyalrongic*** *languages in the database*. Full explicitness would be a service to some readers: adding a clarification to the effect that ‘(Bantawa and Old Burmese, which are not Rgyalrongic languages, are not shown on this map.)’ Some redundancy would help here. The profusion of proper names specific to the area, and the presence of concepts specific to historical linguistics, are perfectly natural in work of this nature, and are essentially inevitable. But their combination tends to put off members of an extended readership of linguists, when one’s best efforts at imbibing the area-specific information in an article leave one baffled. So, efforts at clarity are most needed, to accompany readers accustomed to softer linguistic landscapes. (Decisions at the authors’ choice.) The issue extends to language names and language identifiers. There is room for improvement here, and a small amount of further work in this space would entail large benefits for the legibility of the article – and arguably, for the usability of the database for a not-too-narrow public. Currently, language names do not have a unified syntax. Taking «Menya-Gao» as an example, where «Menya» is the language name, and «Gao» the family name of the author who collected the original data: «Menya-Gao» is OK as an identifier (for computational purposes), but not too great as a language name. The syntax is, to say the least, unusual. It could make sense if the authors wanted to lay emphasis on the specific ‘doculects’ used in their study, and to give credit to the authors who provided the indispensable basis for the database, and who bear responsibility for data quality. If so, the syntax would yield: Menya Gao, Muya Huang, Situ Zhang, Japhug Jacques, Stau Gates, Khroskyabs Lai, Rma Sims, Zbu Gong, and so on. If the authors wish to float this new practice, it should be applied consistently. But since information on the source of data is provided in the table, it would make excellent sense to go by common practices in English-language publications, and use a syntax such as:

 In detail, «Kangding Muya» would be somewhat under-specific, since «Menya-Gao» is spoken in Kangding, too, and the main text describes «Muya» and «Menya» as alternative names for Minyag: «Minyag (aka. Muya, Menya)», seemingly in the same way as «Sino-Tibetan» and «Trans-Himalayan» are different labels for the same language family, or, at the language level, «Pumi» and «Prinmi», «Na» and «Mosuo», «Standard Mandarin» and «Putonghua», «Shixing» and «Xumi», etc. But crucially, there *is* a dialect difference between the scopes of the two labels as used in the database: Figure 1 has Muya and Menya as distinct data points. Changes to the language names in Table 1 do not imply any modifications to the suite of computer files & scripts: only a change to the table and to the main text of the presentation article under review here.

 The repository where the dataset is made available could do with quick additional UX improvements, too: improving the experience of less advanced users – basically, linguists with an awareness of the usefulness of computer scripts but without regular practice of scripting. To begin with, a path to the main reference files should be indicated clearly on the repo’s landing page, and perhaps repeated in some of the subfolders, along the lines of ‘This folder contains . In case you are looking for the database in CSV format, please refer to .’ Thus, in the presentation text (the article under review) the language ‘MazurStau’ appears in Figure 1 (as ‘Mazur’) and in Table 1. The forms.csv file, which (at the time of this review) had been updated on April 26th, 2023, has the MazurStau forms. But this reviewer has not been able to locate any ‘MazurStau’ data in the list of cognates (wordlist-cognates.tsv) in the ‘analysis’ folder. For instance: for ‘nose’, the .tsv file has 21 entries; the Stau form /sni/ (Gates 2021:50) is conspicuously cognate, and would make for a complete cognate set of 22 varieties. Is the .tsv file, last updated on April 20th, 2020, an old file, not relevant to the database in the present state? If so, some cleanup of the GitHub repo or additional explanations would be a service to potential users, so they don’t get led down a garden path when following the enticing lead of an ‘analysis’ folder name. Providing such folders with a short README file would constitute sufficient guidance, and would be well worth the effort – pending the release of future newfangled tools on the GitHub web interface that allow for easy navigation to key files: those that are updated most often, that are referenced at key points in scripts, and other such criteria.

 Minor points:

 Introduction: «In order to infer the phylogenetic subgrouping of Rgyalrongic, a lexical dataset with high quality curation is inevitable»: inevitable > indispensible

 In the discussion of elicitation, the mention «without physical contact» is puzzling. What are the intended implications? Does it have to do with the differences in legal frameworks for experiments involving human subjects depending on the protocol, with/without physical contact? If the mention is intended as a legal disclaimer, the statement should be made in a relevant section or footnote, not in the main text, where it acts as a distractor. I would suggest making a reference to a publication that sets out the essentials of linguistic fieldwork, with any qualifications and additions the authors may wish to make. Thus, interested readers would get a useful pointer to a resource explaining about linguistic fieldwork, and broaching the all-important human topic of the investigator’s relationship with consultants.

 p. 5 German *fuß*: why not capitalize the F, thus: *Fuß*?

 Acknowledgements: and detailed explanations to our questions > and detailed explanations in answer to our questions

 References: the URL provided for

 Gates JP: *Grammaire du stau de Mazur*. Paris: Écoles des Hautes Études en Sciences Sociales dissertation, 2021.

 is unhelpful: it is a link to an online announcement of the PhD defense, not a link to the document itself. Since the dissertation is available online, the authors should cite an institutional repository: either the dissertation’s entry on theses.fr ( https://theses.fr/2021EHES0054), or a direct link to the PDF, though the latter option is likely to prove less ‘time-proof’ ( https://www.theses.fr/2021EHES0054/abes/Gates_Jesse_these_2020.pdf).

Are sufficient details of methods and materials provided to allow replication by others?

Partly

Is the rationale for creating the dataset(s) clearly described?

Yes

Are the datasets clearly presented in a useable and accessible format?

Yes

Are the protocols appropriate and is the work technically sound?

Yes

Reviewer Expertise:

Naish subgroup of Sino-Tibetan (Naxi, Na/Mosuo, Laze); phonetics/phonology; language documentation and conservation; Computational Language Documentation

I confirm that I have read this submission and believe that I have an appropriate level of expertise to confirm that it is of an acceptable scientific standard, however I have significant reservations, as outlined above.

# Response

**Published:** 2023-07-08

**Authors:** Yunfan Lai

**Affiliations:** School of Linguistic, Speech a, The University of Dublin Trinity College, Dublin, Leinster, Ireland

Dear Alexis, Thank you very much for your detailed comments. We will improve the paper accordingly after we have received one more review.

# Response

**Published:** 2023-10-18

**Authors:** Yunfan Lai

**Affiliations:** School of Linguistic, Speech a, The University of Dublin Trinity College, Dublin, Leinster, Ireland

- Differences between our database and the STEDT
  - Another crucial difference between our database and the STEDT is that the first author, Lai Yunfan, is a specialist in Gyalrongic languages, and thus capable of detecting doubtful forms/mistakes in first and second hand sources. Once Lai Yunfan finds a potential error in the sources, he would immediately turn to the authors of the sources or native speakers to ask for verification. This is something the STEDT cannot do: they took nearly everything directly from the sources without significant consideration on their accuracy.

 

- On a slightly critical note, the data note presenting the database does not appear to me to do full justice to the database’s design and applications. In the «Plain language summary», the authors’ description of the database as work «in preparation for future work on historical and evolutionary linguistics» strikes me as unnecessarily modest [...]  Thus, the database under review is part of a growing body of data and tools that possesses demonstrated value to the field of Sino-Tibetan diachronic research. I would therefore suggest rephrasing the relevant passage of the «Plain language summary» from «in preparation for future work on historical and evolutionary linguistics» to «as a tool for research in the field of historical and evolutionary linguistics».
  - We sincerely thank the reviewer's confidence in our database. We find that the reviewer's points are indeed useful and encouraging. We will change the wording in our plain language summary accordingly. 

 

- Concerning matters of form, the data note could do with an additional round of text editing to ensure best readability for authors outside the first circle of specialists of this area of Sino-Tibetan. There are 20 languages in Figure 1, and 22 in Table 1. It would be useful to provide clarifications in the caption to Figure 1. Quick fix: Figure 1. Geographical distribution of the Rgyalrongic languages in the database. Full explicitness would be a service to some readers: adding a clarification to the effect that ‘(Bantawa and Old Burmese, which are not Rgyalrongic languages, are not shown on this map.)’ 
  - We have ameliorated the explicitness of the descriptions throughout the paper. 

 

- The issue extends to language names and language identifiers. There is room for improvement here, and a small amount of further work in this space would entail large benefits for the legibility of the article – and arguably, for the usability of the database for a not-too-narrow public. Currently, language names do not have a unified syntax. Taking «Menya-Gao» as an example, where «Menya» is the language name, and «Gao» the family name of the author who collected the original data: «Menya-Gao» is OK as an identifier (for computational purposes), but not too great as a language name [...]
  - The reviewer here suggests a crucial revision. We totally agree with it and made the syntax of language names consistent. 

 

- The repository where the dataset is made available could do with quick additional UX improvements, too: improving the experience of less advanced users – basically, linguists with an awareness of the usefulness of computer scripts but without regular practice of scripting [...]
  - This is indeed a very good suggestion. We will surely try our best to improve the user-friendly-ness. For the time being, we will provide a stable Edictor link accessible for readers and users. Edictor is easy to use with a brief instruction, has a good UX design, and is constantly improving with additional features. 

 

- The forms.csv file, which (at the time of this review) had been updated on April 26th, 2023, has the MazurStau forms. But this reviewer has not been able to locate any ‘MazurStau’ data in the list of cognates (wordlist-cognates.tsv) in the ‘analysis’ folder. For instance: for ‘nose’, the .tsv file has 21 entries; the Stau form /sni/ (Gates 2021:50) is conspicuously cognate, and would make for a complete cognate set of 22 varieties. Is the .tsv file, last updated on April 20th, 2020, an old file, not relevant to the database in the present state? If so, some cleanup of the GitHub repo or additional explanations would be a service to potential users, so they don’t get led down a garden path when following the enticing lead of an ‘analysis’ folder name.
  - This is indeed an important point. The most recent .tsv files are actually in the folder “hacks”, where we have a Python script add_stau.py to add data of Mazur Stau. The resulting .tsv file is rgyalrong-cogid.tsv which includes Mazur Stau. The file wordlist-cognates.tsv is not relevant anymore. We have done a clean-up in our repository. 

 

- Introduction: «In order to infer the phylogenetic subgrouping of Rgyalrongic, a lexical dataset with high quality curation is inevitable»: inevitable > indispensible
  - We have corrected it. 

 

- In the discussion of elicitation, the mention «without physical contact» is puzzling. What are the intended implications? Does it have to do with the differences in legal frameworks for experiments involving human subjects depending on the protocol, with/without physical contact? If the mention is intended as a legal disclaimer, the statement should be made in a relevant section or footnote, not in the main text, where it acts as a distractor. I would suggest making a reference to a publication that sets out the essentials of linguistic fieldwork, with any qualifications and additions the authors may wish to make. Thus, interested readers would get a useful pointer to a resource explaining about linguistic fieldwork, and broaching the all-important human topic of the investigator’s relationship with consultants.
  - This is related to the ethical regulations of our grants. I have made this more explicitly explained to readers. The disclaimers are now in Footnote 1 in the revised version.

 

- p. 5 German fuß: why not capitalize the F, thus: Fuß?
  - We have capitalized it. 

 

- Acknowledgements: and detailed explanations to our questions > and detailed explanations in answer to our questions
  - We have corrected it.
